# Application of Magnetic Materials Combined with Echo^®^ Mass Spectrometry System in Analysis of Illegal Drugs in Sewage

**DOI:** 10.3390/molecules29092060

**Published:** 2024-04-29

**Authors:** Feiyu Yang, Kaijun Ma, Yichao Cao, Zhiyuan Li

**Affiliations:** 1 Shanghai Research Institute of Criminal Science and Technology, Shanghai Key Laboratory of Crime Scene Evidence, Shanghai 200083, China; yccaosh@139.com; 2 Shanghai Institute of Forensic Science, Shanghai Key Laboratory of Crime Scene Evidence, Shanghai 200083, China; makaijun@sina.cn; 3 Shanghai AB Sciex Analytical Instrument Trading Co., Ltd., Beijing 100015, China; zhiyuan.li@sciex.com

**Keywords:** analysis technology of drugs in urban sewage, magnetic solid-phase extraction, acoustic ejection mass spectrometry analysis method

## Abstract

The aim of this study is to solve the problems of the complicated pretreatment and high analytical cost in the detection technology of trace drugs and their metabolites in municipal wastewater. A high-performance magnetic sorbent was fsynthesized for the enrichment of trace drugs and their metabolites in wastewater to develop a magnetic solid-phase extraction pretreatment combined with the acoustic ejection mass spectrometry (AEMS) analytical method. The magnetic nanospheres were successfully prepared by magnetic nanoparticles modified with divinylbenzene and vinylpyrrolidone. The results showed that the linear dynamic range of 17 drugs was 1–500 ng/mL, the recovery was 44–100%, the matrix effect was more than 51%, the quantification limit was 1–2 ng/mL, and the MS measurement was fast. It can be seen that the developed magnetic solid-phase extraction (MSPE) method is a good solution to the problems of the complicated pretreatment and analytical cost in the analysis of drugs in wastewater. The developed magnetic material and acoustic excitation pretreatment coupled with mass spectrometry analysis method can realize the low-cost, efficient enrichment, and fast analysis of different kinds of drug molecules in urban sewage.

## 1. Introduction

The analysis of drugs in waste water can be used to assess the drug consumption of specific populations (workplaces, schools, prisons, etc.) in large regions (cities or countries) and small regions, and can be used to obtain trends in the types of drugs consumed or information on new types of drugs through short-term or long-term sampling and testing. The results of the monitoring are of high reference value for the investigation and seizure of drug manufacturing laboratories, the fight against drug crimes, and the early warning of new psychoactive substances [1,2,3,4,5].

Most of the current research on illegal drugs and their metabolites uses active sampling methods, and then the collected water samples are brought back to the laboratory for pretreatment and instrumental analysis [6,7,8,9]. In various laboratories, solid-phase extraction (SPE) is widely used to extract and enrich the drugs in water samples and liquid chromatography–mass spectrometry is commonly used to detect the analytes. In the process of solid-phase extraction, due to the differences in the physical and chemical properties of drugs, different types of solid-phase extraction columns are used, especially hydrophilic, lipophilic reversed-phase adsorption column Oasis HLB^TM^, and cation-exchange solid-phase extraction column Oasis MCX^TM^ [10,11]. However, this technique takes 0.5–3 h to manually process a sample when dealing with urban wastewater samples of about 50 mL to 100 mL, low levels (ng/L)of drug toxins, and complex matrix interferences. Especially in the case of a large number of sewage samples being tested, the SPE method inevitably requires activation, buffer activation, drenching, drying, elution, nitrogen blowing, and volume fixing before entering the chromatographic analysis, which is a considerable consumption of time and economic costs. Therefore, it is difficult for the current conventional pretreatment methods to meet the actual needs of the rapid analysis of trace drugs in urban wastewater, and there is an urgent need to use scientific and efficient pretreatment methods and highly sensitive detection instruments to establish a rapid analytical method [12,13,14,15,16,17,18].

In recent years, magnetic solid-phase extraction (MSPE) has been applied more to the pretreatment of complex samples in the fields of the environment, food, and biomedicine. Due to its advantages of a simple operation, short extraction time, low use of organic reagents, strong adsorption capacity, and easy automation, MSPE has attracted the attention of many analytical researchers and has achieved very good application results [19,20,21,22]. MSPE has three main advantages: first, it makes the extraction process simple, for it does not require expensive equipment, and the magnetic sorbents can be separated in a short time to adsorb trace analytes in a sample with the volume more than 50 mL; second, the amount of chemical substances used in MSPE is relatively reduced, and there is no secondary pollution; and third, it can not only extract the analytes in solution but can also adsorb analytes in suspension. Because the impurities in the sample are generally antimagnetic substances, it can effectively avoid the interference of impurities. Therefore, MSPE is widely applied to the separation and enrichment of samples in the fields of the environment, food, biology, medicine, and so on. However, the one-step adsorption will result in more matrix interference and lower selectivity, which can be compensated for by the chromatograph or mass spectrometer instrument. This study will develop magnetic materials and establish pretreatment and analytical methods for common drugs and their metabolites in wastewater combined a with mass spectrometer instrument.

**Figure 1:**
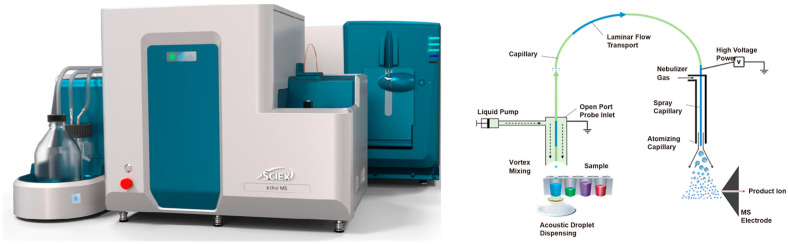
Echo^®^ MS equipment and schematic diagram of the instrument.

Liquid chromatography–mass spectrometry has high specificity and sensitivity and can meet the requirements of scientific applications, especially for samples with a low concentration of analytes [23]. However, liquid chromatography–mass spectrometry analytical techniques usually use gradient elution, which is time-consuming, and the analysis time of one injection may exceed 10–30 min. This study also wants to try a new way to save time on chromatography. The Echo^®^ MS system (Figure 1), Acoustic Ejection Mass Spectrometry (AEMS), is a integrated system consisting of acoustic droplet ejection technology (ADE), an open-port probe sampling interface (OPI), and a powerful quantitative SCIEX Triple Quad™ 6500+ system (with electrospray ionization source). The Echo^®^ MS system is optimized for rapid sampling and analysis at 1 sample per second, which is hundreds of times faster per sample than conventional liquid chromatography mass spectrometry (10 min per sample). The Echo^®^ MS system is compatible with standard 384-well or 1536-well injection plates, which are suitable for rapid, high-throughput sample testing. The Echo^®^ MS system eliminates the need for conventional liquid chromatography consumables such as columns and pre-columns [24,25,26].

Wastewater samples are commonly thought to be difficult to analyze because of their complex matrix. In this study, magnetic solid-phase extraction (MSPE) technology was combined with Echo^®^ MS system detection technology to establish pretreatment and analytical methods for the rapid analysis of drugs and their metabolites in wastewater. In our study, the lack of a chromatographic technique could presumably lead to isobaric interferents and ionization suppression, so we firstly apply magnetic nanoparticles with adsorption for illegal drugs also including other weakly polar and non-polar organics to partly decrease the matrix effects, and secondly make use of the interference resistance of an acoustic mass spectrometer coupled to a QqQ to increase the selectivity. Combining a conventional Echo^®^ MS with high-throughput magnetic solid-phase extraction technology not only saves preprocessing time but also saves analysis time. This study is the first report of the integration of the magnetic solid-phase sample pre-concentration with the Echo^®^ MS system to achieve both high-throughput and high-sensitivity simultaneously.

## 2. Results

### 2.1. Magnetic Solid-Phase Extraction Technique and Characterization of Magnetic Materials

Magnetic solid-phase extraction is a kind of adsorption coating with magnetic particles as the core and the outer surface modified to have a strong adsorption effect on the analyte. In the magnetic solid-phase extraction process, the magnetic adsorbent is not directly filled into the adsorption column but is added to the solution or suspension of the sample; the analyte is adsorbed to the dispersed magnetic adsorbent surface, and under the action of an external magnetic field, the analyte can be separated from the sample matrix.

**Figure 2:**
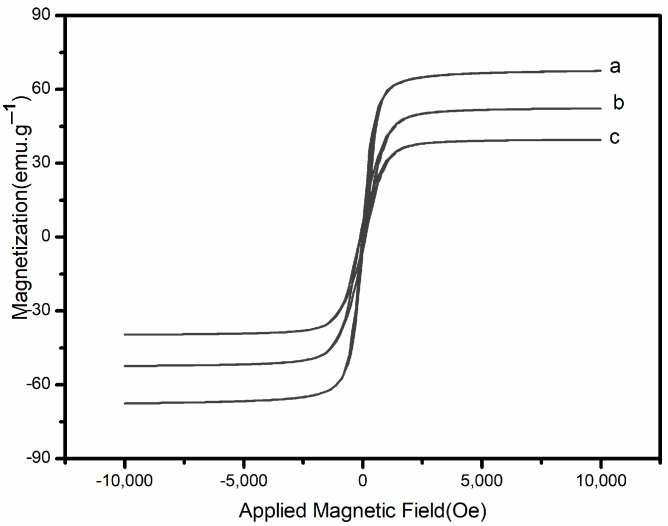
Vibrating sample magnetometer analysis.

Figure 2 shows the hysteresis curve of Fe_3_O_4_, Fe_3_O_4_@SiO_2_-MA, and Fe_3_O_4_@SiO_2_-MA@PLS at room temperature. As can be seen, the three curves have a similar shape and symmetry about the origin. The saturation magnetization value was found to be 52.6 emu·g^−1^ for Fe_3_O_4_@SiO_2_-MA and 67.4 emu·g^−1^ for Fe_3_O_4_. This difference might be attributed to the non-magnetic SiO_2_-MA shell surrounding the magnetite particles. After divinylbenzene and vinylpyrrolidone were grafted on Fe_3_O_4_@SiO_2_-MA, the saturation magnetization value for Fe_3_O_4_@SiO_2_-MA@PLS was 38.7 emu·g^−1^. This indicated the formation of a PLS shell on the surface of the SiO_2_-MA shell.

**Figure 3:**
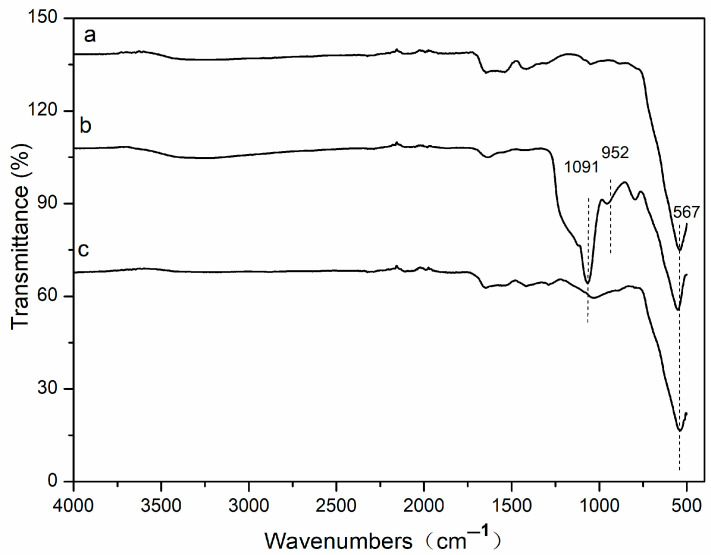
Fourier transform infrared spectroscopy.

Figure 3 shows the Fourier transform infrared spectroscopy that was used to characterize the chemical interaction between Fe_3_O_4_ and functional groups. As can be seen from Figure 3, the adsorption band of Fe-O is at 567 cm^−1^ (Figure 3a), which is the characteristic peak of Fe_3_O_4_ nanoparticles. The two bands at 952 and 1091 cm^−1^ (Figure 3b) are the stretching vibration of Si-O bonds of the SiO_2_ shell. These prove that the SiO_2_ shell is linked to the surface of the magnetic Fe_3_O_4_. In Figure 3c, the peak of 1091 cm^−1^ is almost invisible, and peaks in the region of 1100–1400 cm^−1^ are attributed to C-H and C-C stretching vibrations from divinylbenzene and vinylpyrrolidone. These results indicate that the divinylbenzene and vinylpyrrolidone are successfully chemisorbed on the surface of Fe_3_O_4_@SiO_2_-MA@PLS nanoparticles.

**Figure 4:**
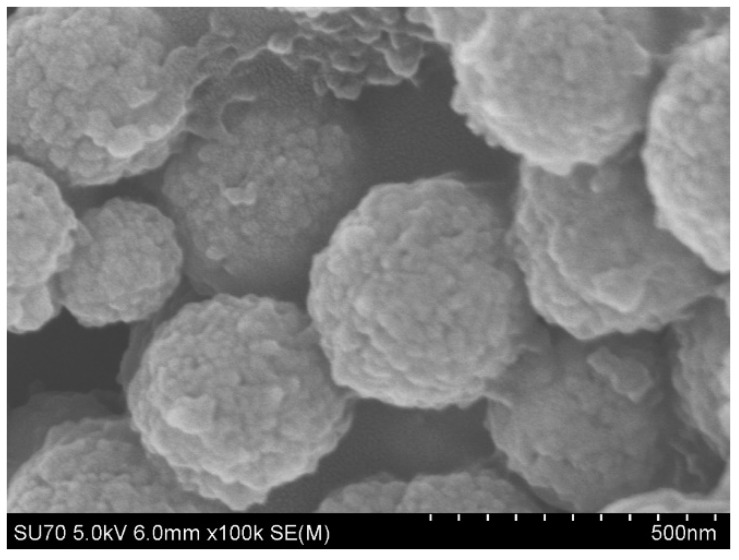
Microstructure of magnetic materials.

The detailed morphological and structural features of the prepared Fe_3_O_4_@SiO_2_-MA@PLS nanoparticles were characterized using SEM. Figure 4 indicates that nanoparticles are well-dispersed with the average size of 200 nm.

### 2.2. Method Validation

**Figure 5:**
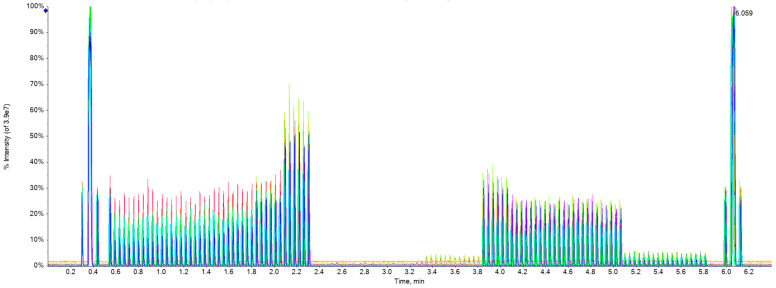
Total chromatograms showing analysis for this study.

Figure 5 is a representative synthetic chart created by Echo^®^ MS system software (SCIEX OS 1.6.1) overlaying the chromatograms with 17 colors together obtained by the corresponding methods of 17 drugs in one experiment turn (not the results of all samples), where each method takes about 6 min and the total of all methods takes 6 × 17 = 102 min. For each analyte method, 0.6~2.3 min was for linear analysis, 2.4~3.2 min was for blank samples, and 3.3~5.8 min was for recovery and matrix effect analysis.

The linear range, the linear regression equation, the coefficient, and the LOQs are shown in Table 1, and demonstrate that the linear range of the 17 drugs was 1–500 ng/mL, and the limit of quantification was 1–2 ng/mL.

**Figure 6:**
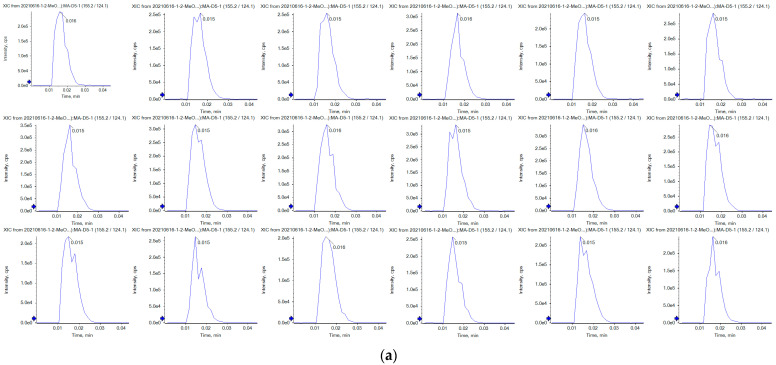

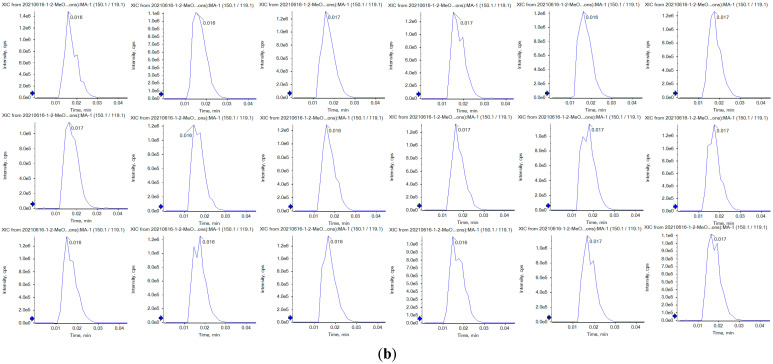
Chromatograms of methamphetamine-D5 (**a**) and methamphetamine in Sample 5, Sample 6, Sample 7 (6 consecutive injections) (**b**).

The precision results indicated that the RSD values of the peak areas of the 17 drugs were less than 5%, and the detailed data are shown in Table 2 below. Figure 6a,b show typical chromatograms of methamphetamine-D5 and methamphetamine in Sample 5, Sample 6, Sample 7 for six consecutive injections. The examination of the matrix effects and extraction recovery of the 17 drugs are listed in Table 3, which shows that this MDSPE method had an acceptable matrix effect and recovery except for Norketamine and Cathinone.

## 3. Materials and Methods

### 3.1. Material and Reagents

Native drug standards and mass-labeled internal standards were obtained from the third Institute of the Ministry of Public Security (Shanghai, China) and the purity is shown in Supplementary Materials Table S1. Synthetic magnetic bead reagents were purchased from Beijing Chemical Industry Co Ltd. (Beijing, China). All chemicals can be used directly without further purification.

### 3.2. Test Instruments and Analytical Methods

The AEMS system was an Echo^®^ MS system with LC-MS grade water as the coupling fluid, methanol + 0.1% formic acid as the mobile phase, a flow rate of 360 µL/min, and SP mode (i.e., the sample viscosity is less than that of water) as the injection mode. The injection volume was 2.5 nL and the mass spectrometer was a SCIEX Triple Quad™ 6500+ system. The curtain gas was 20 psi, the CAD gas was 9 unit, the ionspray voltage was 5500 V, the source temperature was 300 °C, the ion source gas1 was 90 psi, and the ion source gas2 was 45 psi. MRM transition information is shown in Table 4. Scanning electron micrographs (SEM) were obtained with a S3400N scanning electron microscope (Hitachi, Tokyo, Japan). Infrared spectra were recorded by a Nicolet 6700 FT-IR spectrophotometer (Nicolet, Waltham, MA, USA). The magnetic properties were analyzed through a vibrating sample magnetometer (VSM, PPMS-9) made by Quantum Design, Ltd., San Diego, CA, USA.

### 3.3. Synthesis of Magnetic Adsorbents

Amounts of 1 g of ferric chloride hexahydrate, 3 g of sodium acetate, and 0.2 g of sodium citrate were added to 100 mL of ethylene glycol and stirred to dissolve for 30 min, then added to a 200 mL polytetrafluoroethylene reactor and placed in an oven at 200 °C for 12 h. After being cooled to room temperature, the magnetic separation was washed three times repeatedly using ethanol and deionized water to obtain magnetic tetraferric oxide particles (Fe_3_O_4_). In total, 1 g of 400 nm Fe_3_O_4_ was dispersed in 200 mL of ethanol in ultrasonic treatment, and then 50 mL of water was added to the above dispersion by ultrasonic treatment for 5 min. After stirring for 30 min, 2 mL of concentrated ammonia was added to the solution and mixed thoroughly for 20 min; then 5 mL of tetraethyl orthosilicate and 1.5 mL methacrylic acid-3-(trimethoxymethylsilyl) propyl ester were added drop by drop, and the reaction was continued for 12 h at room temperature to obtain double-bond modified magnetic silica microspheres (Fe_3_O_4_@SiO_2_-MA) [27]. The magnetic separation was cleaned three times with ethanol and deionized water repeatedly, and then the products were placed in a vacuum at 60 °C. The magnetic separation was washed three times with ethanol and deionized water, and particles were dried in a vacuum oven at 60 °C. In total, 2 g of the above particles was dispersed in a 1000 mL round-bottomed flask and added to 500 mL of acetonitrile, and then 3.6 mL of divinylbenzene, 7.2 mL of vinylpyrrolidone, and 0.4 g of 2,2-azobisisobutyronitrile were added to the solution. The mixture was allowed to react for 1 h with mechanical stirring under nitrogen protection. Then, the temperature was set to 75 °C and the mixture was allowed to continue to react at that temperature for 8 h. The magnetic separation was washed three times with deionized water and ethanol, and finally, the product (Fe_3_O_4_@SiO_2_-MA@PLS) was dried at room temperature.

### 3.4. Wastewater Pretreatment Method

**Figure 7:**
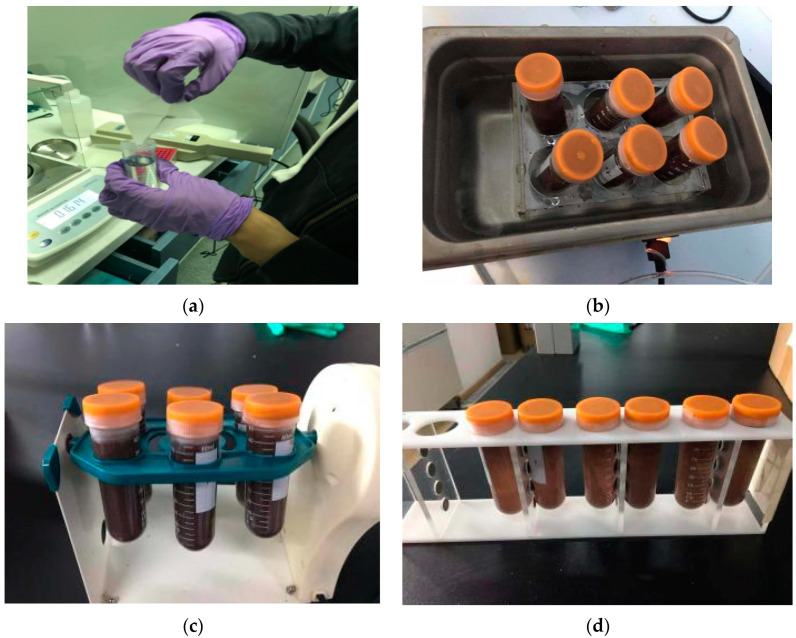
MSPE process of weighing of magnetic materials (**a**), ultrasonic dispersion (**b**), rotary mixing (**c**), magnetic separation (**d**).

As shown in Figure 7, 20 mg of MSPE sorbent was added to a centrifuge tube containing 50 mL of wastewater samples (including mass-labeled internal standards), sonicated for 1 min to fully disperse, and then vortexed at room temperature for 10 min. Subsequently, a magnet was placed on the side of the centrifuge tube in order to separate the MSPE sorbent from the solution. The solution became clear after about 60 s and the supernatant was carefully removed. The analytes were eluted from the magnetic solid-phase extraction adsorbent by ultrasonic washing for 5 min with 3 mL of acetonitrile solution, the eluate was quickly blown dry using nitrogen at 60 °C, re-solubilized using 200 µL of methanol solution (methanol: water = 2:8, *v*/*v*) and passed through a 0.22 µm filter membrane to remove impurities, and finally, injected into the sample for analysis.

### 3.5. Method Validation

Mixed standard solutions were sequentially diluted with methanol water (methanol–water = 2:8, *v*/*v*) as the solvent. Calibration standards in methanol water with concentrations of 1, 2, 5, 10, 20, 50, 100, 250, and 500 ng/mL were prepared for the calibration curves. The concentration of the internal standard contained in each calibration standard solution was 250 ng/mL. By injecting spiked samples at different concentrations, the linearity was investigated based on the peak area ratio of the analytes to the internal standards. The performance of the method was evaluated by linearity, limit of quantification (LOQ), precision, and accuracy. The limit of quantification (LOQ) based on signal-to-noise ratios of 10 (S/N = 10) was determined. Three validation batches were tested to assess the precision of the method.

Samples spiked with 17 analytes at concentrations of 20 ng/mL, 100 ng/mL, and 250 ng/mL were used to investigate the matrix effect and recovery. Three blank matrices provided by different labs at low, medium, and high QC levels (n = 6) were used to evaluate the matrix effect, and the matrix effect was determined as the peak area ratio of the analytes to mass-labeled internal standards when standards were added after extraction divided by the peak area ratio of the analytes to mass-labeled internal standards when standards were added in solvent. Three blank samples were prepared at low, medium, and high QC levels (n = 6) to evaluate the recovery, and the recovery was determined as the peak area ratio of the analytes to mass-labeled internal standards when standards were added before extraction divided by the peak area ratio of the analytes to mass-labeled internal standards when standards were added after extraction. Intra-assay precision was evaluated by the relative standard deviation (RSD) of the measured values of the 3 different samples (i.e., Sample 5, Sample 6, Sample 7) spiked at 250 ng/mL (n = 6) when standards were added before pretreatment.

## 4. Conclusions

In this study, magnetic adsorbents were prepared for the enrichment of drugs and their metabolites in wastewater, and the magnetic dispersive solid-phase extraction (MSPE) pretreatment combined with direct injection mass spectrometry (MS) analysis was established. Magnetic nanomaterials were used as adsorbents with a 250 enrichment factor (50 mL enriched to 200 µL), which were mixed with water, and then concentrated by magnetic separation and elution. It is faster, more economical, less time-consuming, and easier to be high-throughput than the methods of traditional solid-phase extraction. Currently, high-throughput pretreatment with magnetic solid-phase extraction is common for small-volume samples, such as nucleic acid extraction. High-throughput sample preparation equipment for large-volume samples (>5 mL) is not common but is achievable. The magnetic solid-phase extraction automobile equipment with a 24-sample high-throughput developed by our team is in the final stage of testing, and we are performing tests in which coupling with high-throughput direct injection mass spectrometry will dramatically reduce the experiment’s analyzing time. The speed of the analysis make this method a valuable tool compared to traditional methods. However, because there is no chromatographic separation step, the potential isobaric interferences will limit quantification capabilities. Another drawback comes from the ionization suppression effect, which arises from competitive ionization with other components in the matrix.

The Echo^®^ MS system is equipped with ADE technology, which uses acoustic energy to excite the sample from a very small sample volume (2.5 nL) in the sample plate, and the small droplets of the sample are transported to the SCIEX Triple Quad™ 6500+ system for analysis and detection via OPI technology, eliminating the need for conventional liquid chromatography consumables, such as columns and pre-columns, in the entire process. The injection volume of the system is 2.5 nL with dilution during injection, which is 1/1000th of that of a conventional LC-MS/MS. It is very favorable for complex matrices as the negative matrix effects are greatly diluted. At the same time, other substances in the wastewater enriched on the surface of the magnetic beads in the wastewater are also diluted, which also makes the interferences decrease.

The Echo^®^ MS system is equipped with ultra-fast injection speed, 1 sample per second for rapid sampling and analysis, and standard 384-well or 1536-well injection plates, which is suitable for rapid and high-throughput sample detection. In one of our experiments, 127 samples can be completed in 6.3 min, while 1397 min is needed compared with the liquid–mass spectrometry method. The Echo^®^ MS system analyzes the same samples at a rate of at least 220 times faster than conventional liquid–mass spectrometry methods. The Echo^®^ MS system equipped with the SCIEX Triple Quad™6500+ system also possesses powerful quantification capability, and the data showed that the limit of quantification (LOQ) for the 17 drugs could reach 1–2 ng/mL. In the precision study, the Echo^®^ MS system was able to achieve the RSD of matrix samples of <5%. In addition, the recovery of all 17 drugs could reach more than 80% (except Norketamine and Cathinone) in the investigation experiment. The methodological data showed that the proposed method could provide a promising application for the analysis of drugs and their metabolites in complex water samples.

## Figures and Tables

**Table 1:** Linear range and limit of quantification for 17 drugs.

No	Drugs	Linear Range (ng/mL)	LOQ (ng/mL)	Linear Regression Equation	R^2^
1	Amphetamine	2–500	2	y = 0.00200 x + −8.92197e^−4^	0.99968
2	Methamphetamine	1–250	1	y = 0.00625 x + 0.00113	0.99603
3	O6-monoacetylmorphine	2–500	2	y = 0.00463 x + 3.36531e^−4^	0.99827
4	Morphine	2–500	2	y = 0.00218 x + 1.56023e^−4^	0.99509
5	Ketamine	1–500	1	y = 0.00318 x + 0.00102	0.99772
6	Norketamine	2–500	2	y = 0.00256 x + 0.00136	0.99593
7	Cocaine	1–500	1	y = 0.00721 x + 0.00269	0.99694
8	Benzoylecgonine	1–500	1	y = 0.00299 x + 0.00105	0.99501
9	3,4-Methylenedioxyamphetamine	2–500	2	y = 0.04854 x + −0.00804	0.99952
10	3,4-methylenedioxymethamphetamine	2–500	2	y = 0.00244 x + −5.97610e^−4^	0.99522
11	Cathinone	1–500	1	y = 0.00341 x + 0.00402	0.99550
12	Methcathinone	1–500	1	y = 0.00162 x + −0.00110	0.99264
13	Fentanyl	1–500	1	y = 0.00608 x + −0.00142	0.99910
14	Diazepam	1–500	1	y = 0.00313 x + 0.00316	0.99822
15	Estazolam	1–500	1	y = 0.00394 x + 5.11284e^−4^	0.99747
16	Methadone	1–500	1	y = 0.00664 x + 0.00565	0.99431
17	N-(1-methoxy-3,3-dimethyl-1-oxobutan-2-yl)-1-(5-fluoropentyl)-1H-indole- 3-carboxamide	1–500	1	y = 0.02089 x + 0.03709	0.99773

**Table 2:** Intra-assay precision of samples spiked at 250 ng/mL by MSPE.

No.	Drugs	Precision, RSD % (n = 6)		
		Sample 5	Sample 6	Sample 7
1	Amphetamine	3.4	2.3	2.5
2	Methamphetamine	3.4	3.8	4.5
3	O6-monoacetylmorphine	2.7	3.4	3.4
4	Morphine	1.6	4.9	4.7
5	Ketamine	2.6	2.7	3.7
6	Norketamine	4.0	0.8	4.8
7	Cocaine	4.8	4.8	3.6
8	Benzoylecgonine	1.4	2.5	2.7
9	3,4-Methylenedioxyamphetamine	2.3	3.4	4.1
10	3,4-methylenedioxymethamphetamine	4.4	3.4	3.6
11	Cathinone	1.9	4.3	3.7
12	Methcathinone	2.5	4.1	2.9
13	Fentanyl	4.2	4.6	4.9
14	Diazepam	3.3	4.4	4.5
15	Estazolam	2.1	3.3	1.9
16	Methadone	5.0	4.9	2.3
17	N-(1-methoxy-3,3-dimethyl-1-oxobutan-2-yl)-1-(5-fluoropentyl)-1H-indole- 3-carboxamide	1.1	3.7	2.0

**Table 3:** Matrix effects and recoveries for 17 drugs.

No.	Drugs	Matrix Effects % (20 ng/mL) ^a^ (%RSD)	Recoveries % (20 ng/mL) ^a^ (%RSD)	Matrix Effects % (100 ng/mL) ^b^ (%RSD)	Recoveries % (100 ng/mL) ^b^ (%RSD)	Matrix Effects % (250 ng/mL) ^c^ (%RSD)	Recoveries % (250 ng/mL) ^c^ (%RSD)
1	Amphetamine	75 (7.1)	85 (4.6)	83 (5.7)	96 (3.1)	88 (6.3)	95 (2.9)
2	Methamphetamine	80 (6.9)	90 (5.2)	89 (6.1)	91 (4.9)	95 (4.3)	92 (3.5)
3	O^6^-monoacetylmorphine	84 (7.2)	89 (6.2)	92 (6.3)	97 (5.6)	100 (4.9)	99 (5.8)
4	Morphine	79 (8.5)	92 (7.9)	91 (7.2)	97 (5.1)	94 (6.1)	97 (4.3)
5	Ketamine	81 (3.9)	88(3.2)	89 (4.1)	96 (6.3)	91 (3.5)	97 (5.4)
6	Norketamine	82 (8.2)	59 (3.9)	72 (7.8)	67 (7.5)	69 (4.6)	68 (6.3)
7	Cocaine	91 (3.1)	90 (2.9)	93 (4.2)	92 (3.7)	96 (2.3)	93 (1.6)
8	Benzoylecgonine	87 (3.9)	91 (3.1)	84 (2.8)	99 (2.2)	91 (6.4)	95 (4.1)
9	3,4-Methylenedioxyamphetamine	83 (5.6)	87 (3.5)	87 (3.4)	87 (3.1)	92 (4.7)	97 (6.7)
10	3,4-Methylenedioxymethamphetamine	86 (4.8)	83 (3.9)	92 (3.6)	94 (4.8)	91 (5.3)	88 (6.3)
11	Cathinone	67 (9.7)	46 (8.5)	69 (7.2)	56 (7.5)	51 (6.8)	44 (4.3)
12	Methcathinone	63 (7.3)	86 (6.6)	108 (8.9)	82 (6.4)	107 (6.2)	95 (5.2)
13	Fentanyl	86 (3.4)	80 (3.6)	86 (5.1)	93 (4.3)	84 (6.9)	90 (3.1)
14	Diazepam	92 (4.7)	93 (2.5)	95 (4.3)	100 (3.6)	101 (5.1)	95 (4.7)
15	Estazolam	89 (6.3)	93 (2.9)	79 (3.2)	83 (5.1)	89 (3.3)	91 (2.2)
16	Methadone	77 (7.4)	88 (6.2)	90 (6.6)	95 (4.9)	87 (5.6)	92 (7.8)
17	N-(1-methoxy-3,3-dimethyl-1-oxobutan-2-yl)-1-(5-fluoropentyl)-1H-indole- 3-carboxamide	78 (5.9)	89 (5.5)	85 (7.8)	91 (4.6)	83 (3.4)	96 (6.1)

^a^ Spiked at 20 ng/mL; ^b^ spiked at 100 ng/mL; ^c^ spiked at 250 ng/mL.

**Table 4:** MRM transition information of 17 kinds of drugs and their deuterium compounds.

No.	Drugs	Precursor Ion (*m*/*z*)	Fragment Ion (*m*/*z*)	Declustering Potential (V)	Collision Energy (V)
1	amphetamine	136.1	119.1 *	20	13
		136.1	91.1	20	23
	amphetamine-D5	141.1	124.1 *	20	13
2	methamphetamine	150.1	119.1 *	25	16
		150.1	91.1	25	27
	methamphetamine-D5	155.2	121.1 *	25	16
3	O^6^-monoacetylmorphine	328.2	211.1 *	120	34
		328.2	165.1	120	50
	O^6^-monoacetylmorphine-D3	331.2	211.1	120	34
4	morphine	286.1	201.1 *	110	36
		286.1	165.1	110	57
	morphine-D3	289.2	201.1 *	110	36
5	ketamine	238.1	207.1 *	35	19
		238.1	125	35	35
	ketamine-D4	242.1	211.1 *	35	19
6	Norketamine	224.1	207.1 *	30	18
		224.1	125	30	35
	Norketamine-D4	228.1	211.1 *	30	18
7	Cocaine	304.2	182.1 *	80	27
		304.2	150.1	80	32
	Cocaine-D3	307.2	185.1 *	80	27
8	Benzoylecgonine	290.1	168.1 *	70	26
		290.1	105	70	36
	Benzoylecgonine-D3	293.1	171.1 *	70	26
9	3,4-Methylenedioxyamphetamine	180.1	133.1 *	15	25
		180.1	105.1	15	30
	3,4-Methylenedioxyamphetamine-D4	184	167 *	15	16
10	3,4-methylenedioxymethamphetamine	194.1	163.1 *	30	16
		194.1	105.1	30	32
	3,4-methylenedioxymethamphetamine-D4	198.1	167.1 *	30	16
11	Cathinone	150.4	117.2 *	30	31
		150.4	132.2	30	17
	Cathinone-D5	155.3	122 *	40	31
12	Methcathinone	164.1	105.1 *	70	31
		164.1	131.1	70	26
	Methcathinone-D5	169.1	136.1 *	70	31
13	Fentanyl	337.2	188.3 *	90	31
		337.2	105.2	90	45
	Fentanyl-D5	342.2	105 *	90	45
14	Diazepam	285.1	193 *	125	44
		285.1	154	125	35
	Diazepam-D5	290	198 *	125	44
15	Estazolam	295.2	267.3 *	130	32
		295.1	205.2	130	54
	Estazolam-D5	300	272 *	130	34
16	methadone	310.2	265.2 *	40	21
		310.2	105.1	40	34
	methadone-D10	320	275 *	40	21
17	N-(1-methoxy-3,3-dimethyl-1-oxobutan-2-yl)-1-(5-fluoropentyl)- 1H-indole-3- carboxamide(5F-MDMB-PICA)	377	232 *	110	20
		377	144	110	54
	N-(1-methoxy-3,3-dimethyl-1-oxobutan-2-yl)-1-(5-fluoropentyl)- 1H-indole- 3-carboxamide-D4	381	236 *	110	20

* Quantifier ion.

## Data Availability

The data presented in this study are available on request from the corresponding author.

## Supplementary Materials

The following supporting information can be downloaded at: https://www.mdpi.com/article/10.3390/molecules29092060/s1.
