# Supplementary material for: Application of Magnetic Materials Combined with Echo® Mass Spectrometry System in Analysis of Illegal Drugs in Sewage
Source: Molecules. 2024 Apr 29;29(9):2060. doi: 10.3390/molecules29092060 (PMC11085165; doi:10.3390/molecules29092060)
Supplement: Supplementary file 1 [file molecules-29-02060-s001.zip › molecules-2923778-supplementary.pdf]

Application of magnetic materials combined with Echo® MS system in ultra-fast and ultra-sensitive detection of illegal drugs in sewage

Feiyu Yang[1]*, Kaijun Ma[2], Yichao Cao [1] and Zhiyuan Li[3]

1 Shanghai Research Institute of Criminal Science and Technology, Shanghai Key Laboratory of Crime Scene Evidence, Shanghai 200083, China; yccaosh@139.com
2 Shanghai Institute of Forensic Science, Shanghai Key Laboratory of Crime Scene Evidence, Shanghai 200083, China; makaijun@sina.cn
3 Shanghai AB Sciex Analytical Instrument Trading Co., Ltd., Beijing 100015, China; zhiyuan.li@sciex.com
* Correspondence: yangfyhit@sina.com

Amphetamine

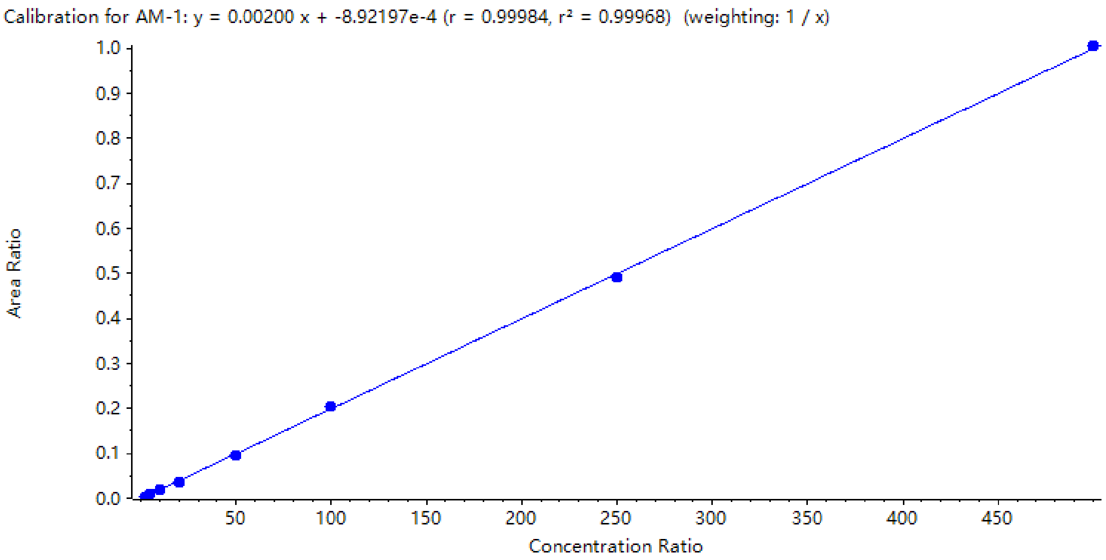

Figure S1. Calibration graph of Amphetamine

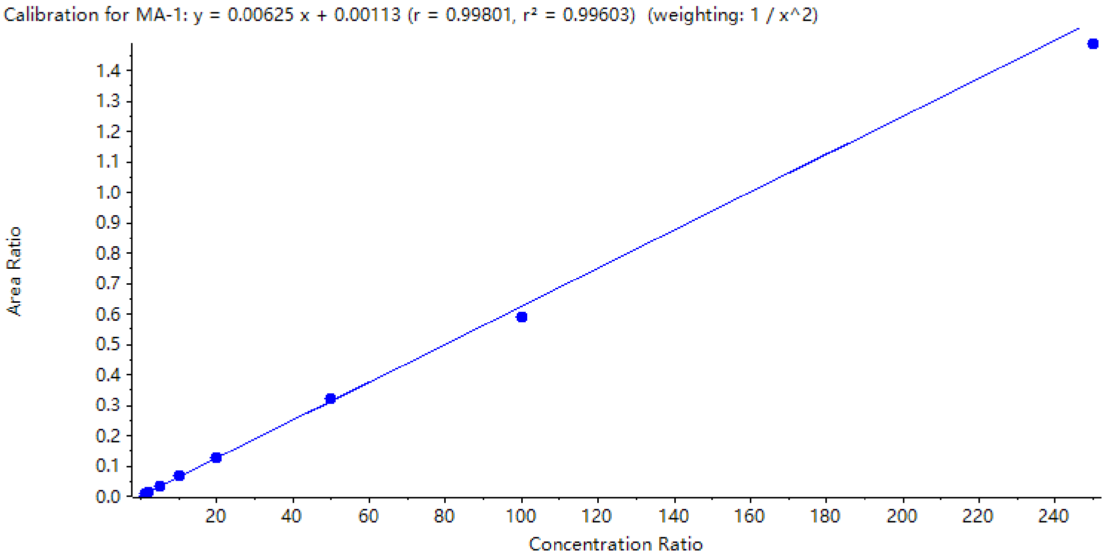

Figure S2. Calibration graph of Methamphetamine

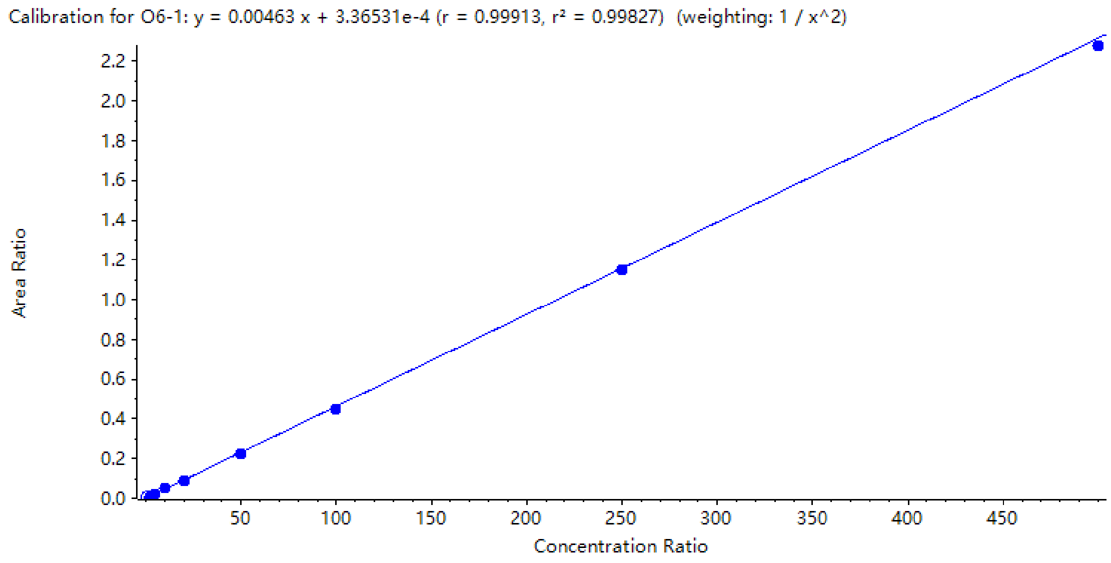

Figure S3. Calibration graph of O6-monoacetylmorphine

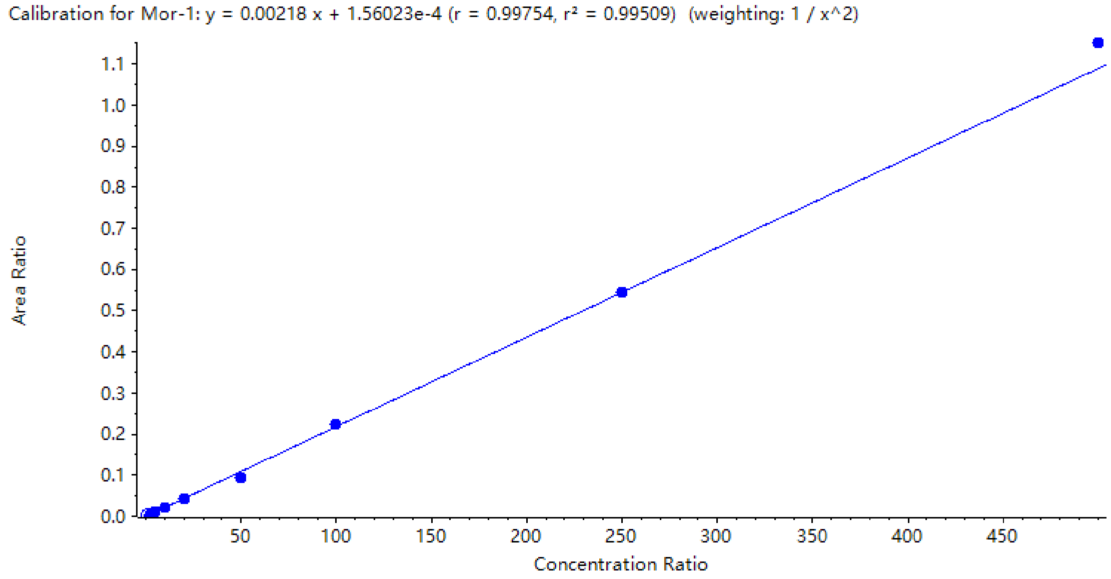

Figure S4. Calibration graph of Morphine

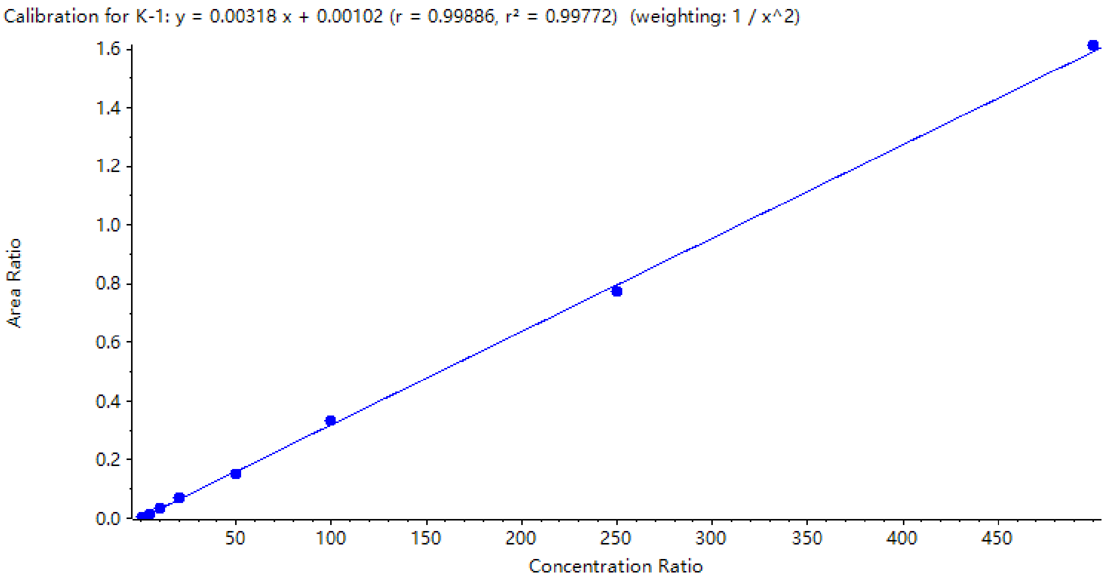

Figure S5. Calibration graph of Ketamine

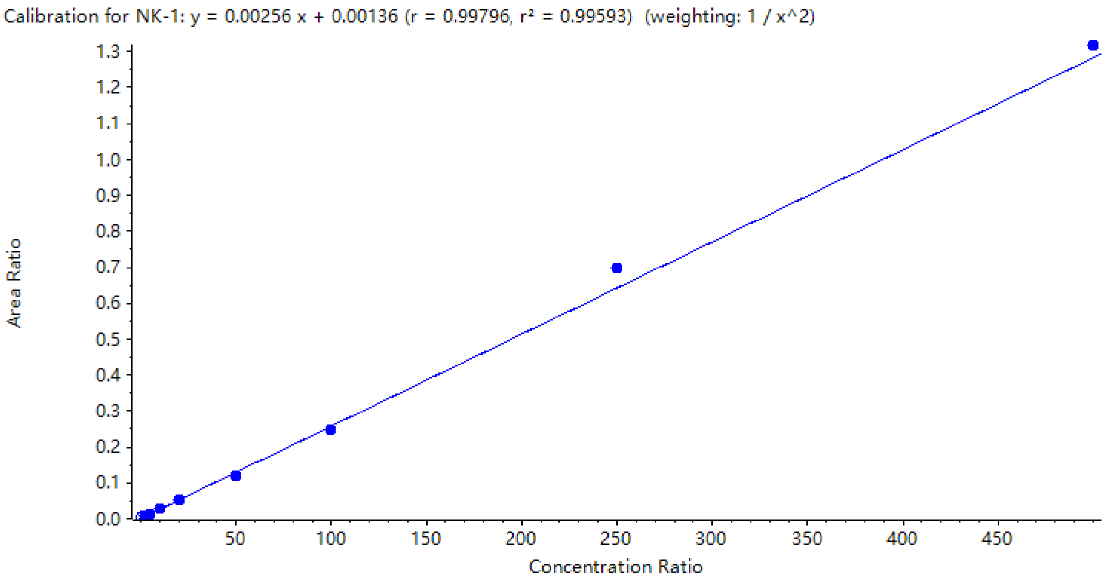

Figure S6. Calibration graph of Norketamine

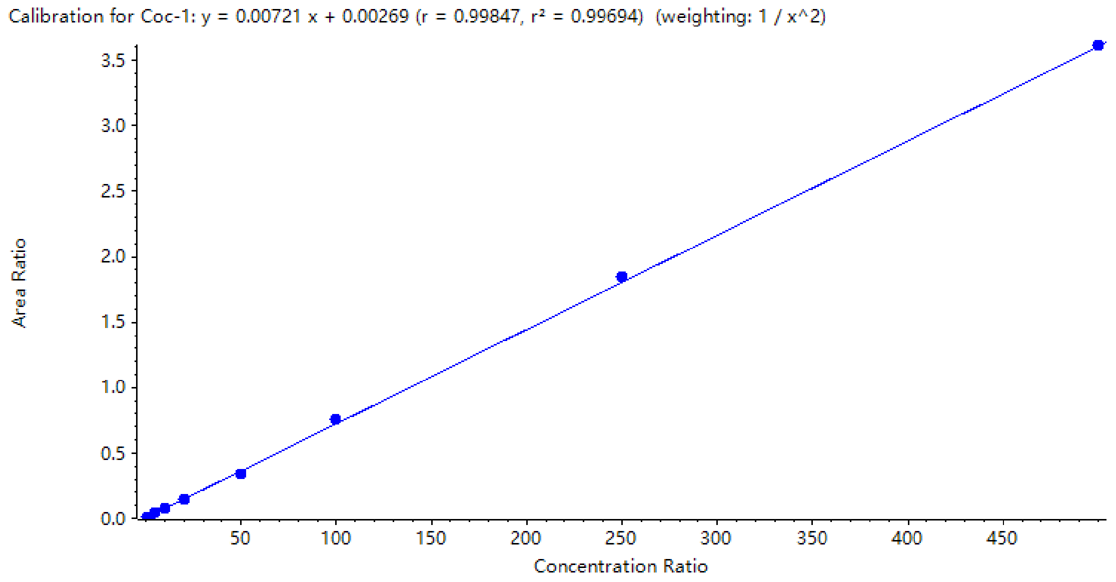

Figure S7. Calibration graph of Benzoylecgonine

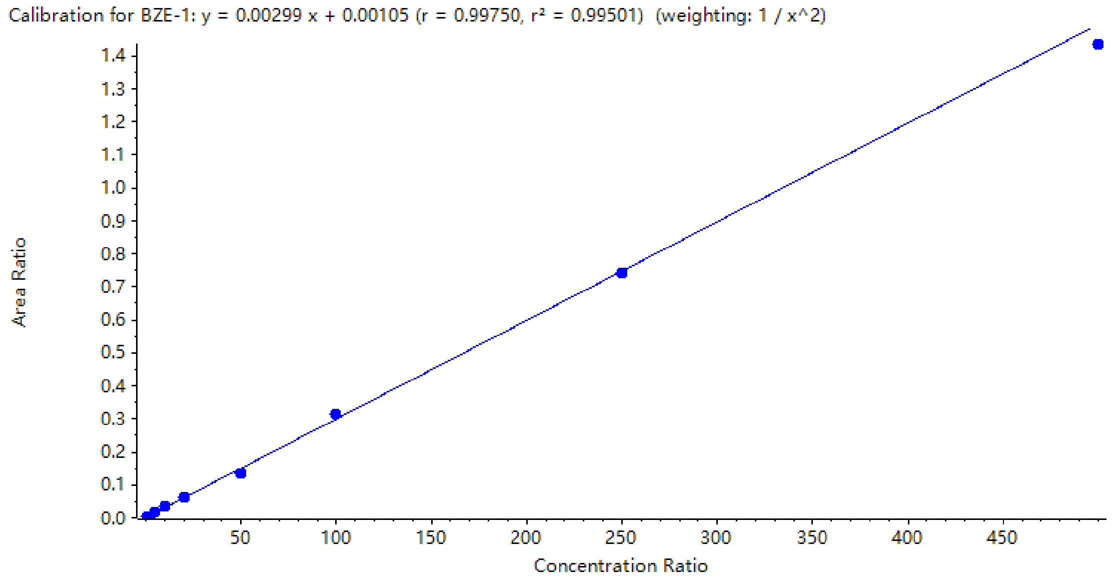

Figure S8. Calibration graph of Benzoylecgonine

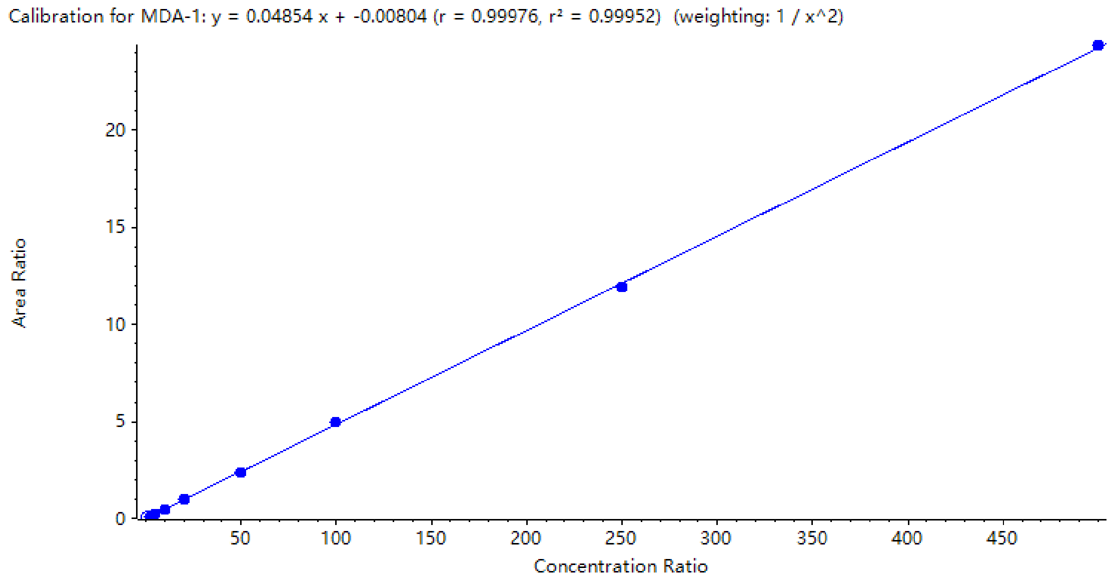

Figure S9. Calibration graph of 3,4-Methylenedioxyamphetamine, MDA

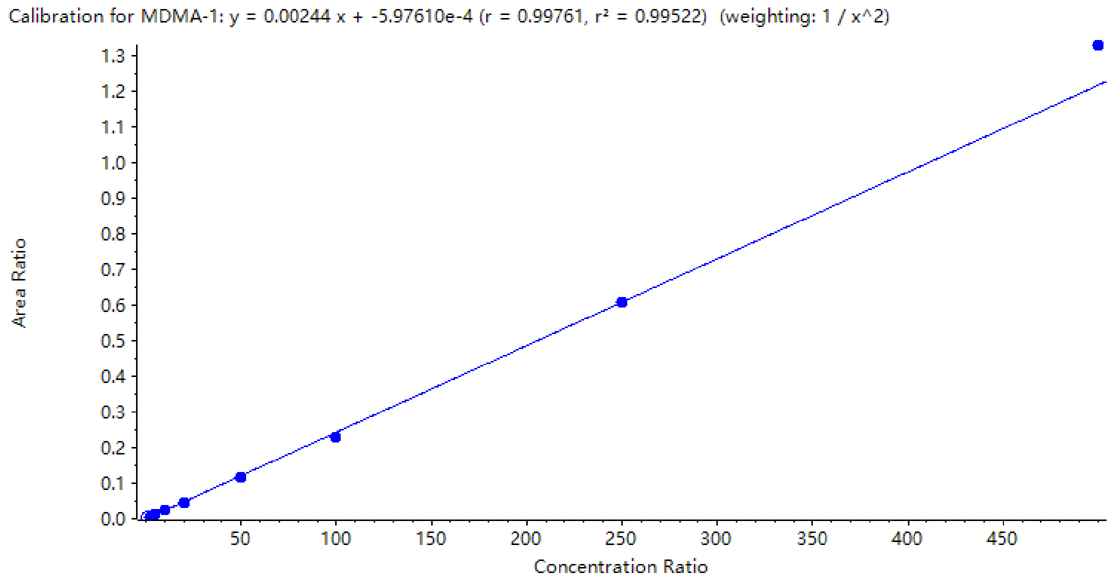

Figure S10. Calibration graph of MDMA

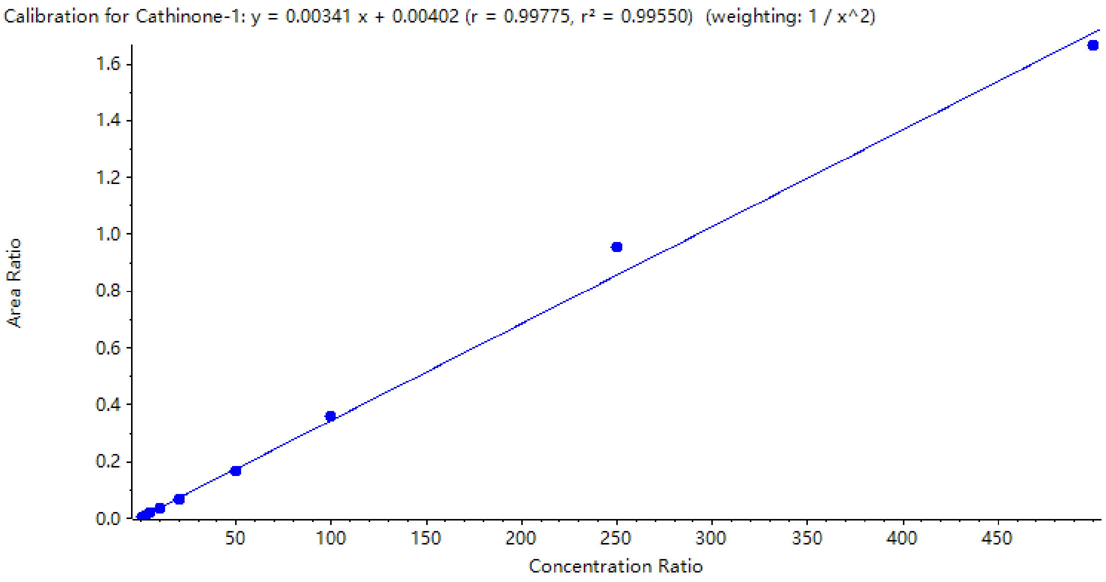

Figure S11. Calibration graph of Cathinone

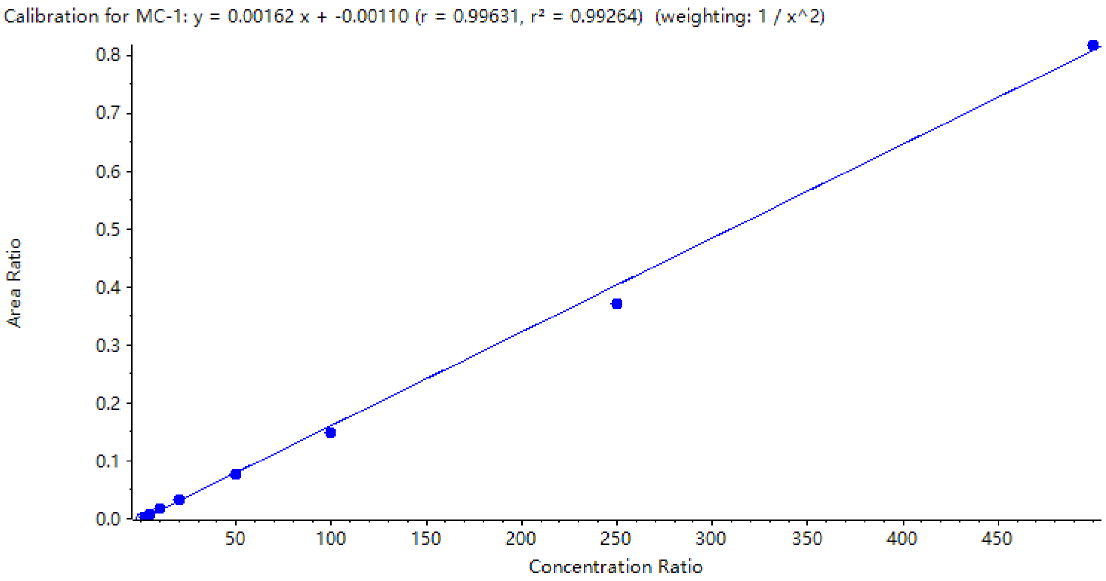

Figure S12. Calibration graph of Methcathinone

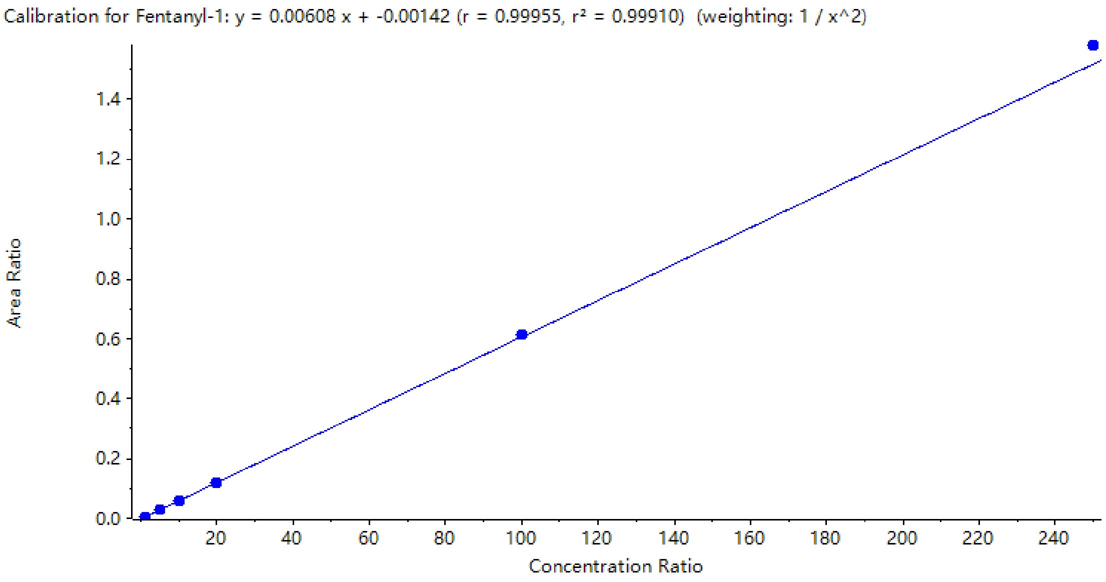

Figure S13. Calibration graph of Fentanyl

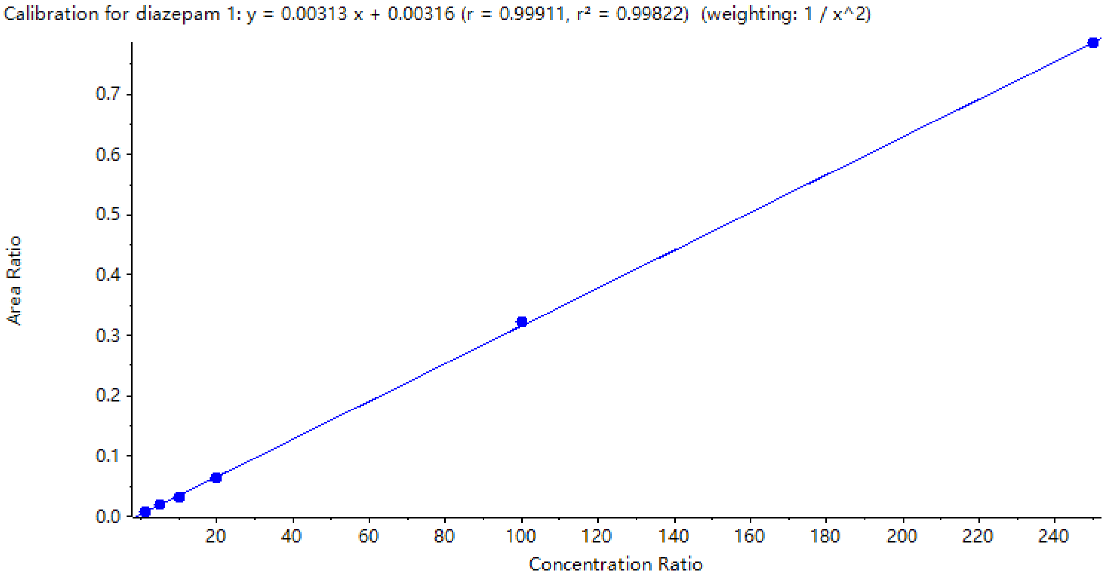

Figure S14. Calibration graph of Diazepam

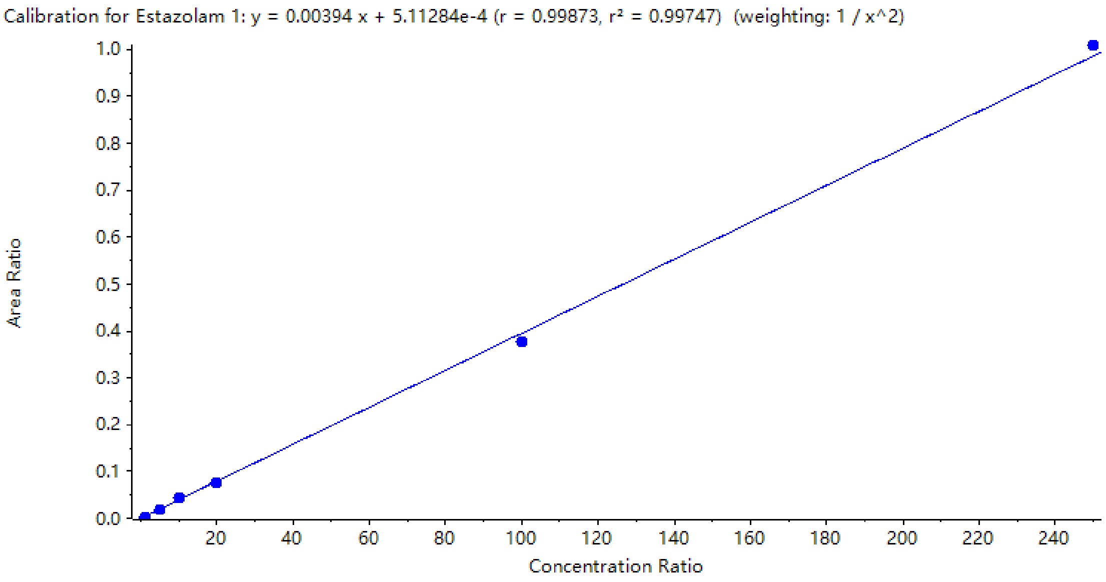

Figure S15. Calibration graph of Estazolam

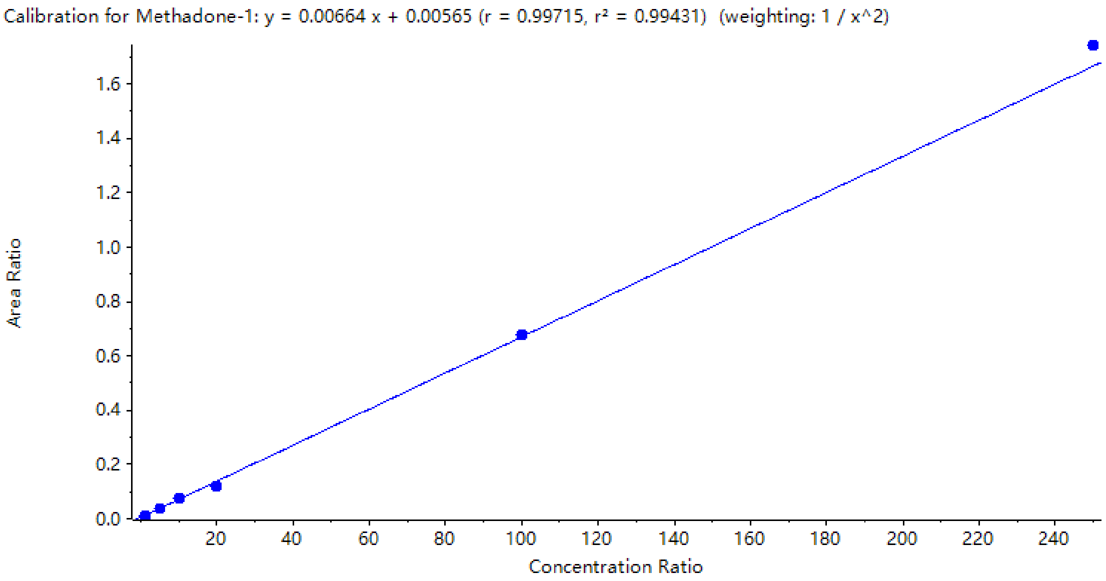

Figure S16. Calibration graph of Methadone

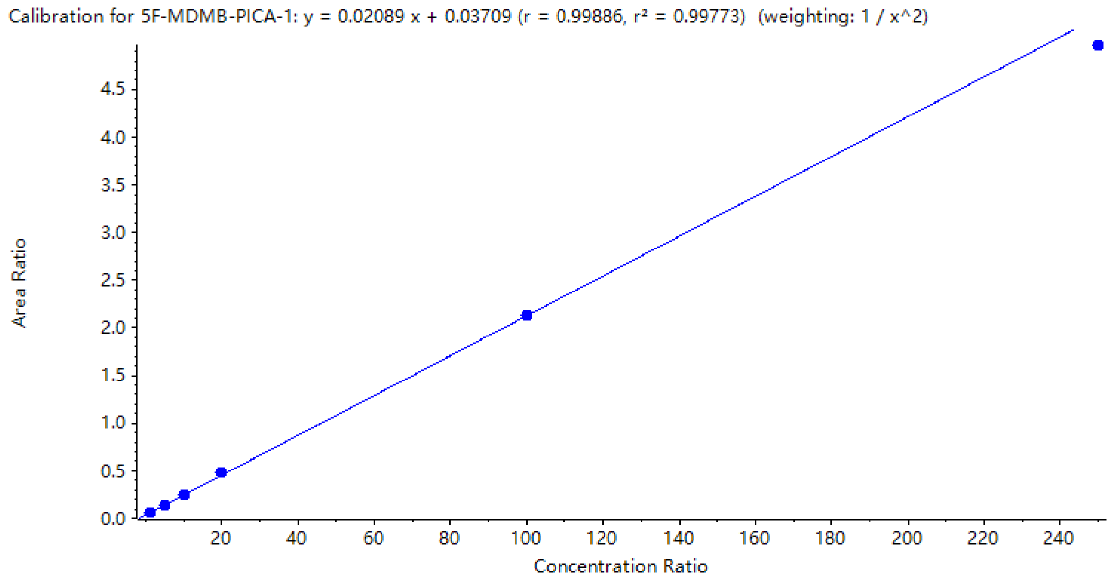

Figure S17. Calibration graph of N-(1-methoxy-3,3-dimethyl-1-oxobutan-2-yl)-1-(5-fluoropentyl)-1H-indole-3-carboxamide

Table S1 The purity of each standard (native and mass-labelled)

No.	Drugs	Purity
1	amphetamine	100ug/mL
	amphetamine-D5	100ug/mL
2	methamphetamine	100ug/mL
	methamphetamine-D5	100ug/mL
3	O^6-monoacetylmorphine	100ug/mL
	O^6-monoacetylmorphine-D3	100ug/mL
4	morphine	100ug/mL
	morphine-D3	100ug/mL
5	ketamine	100ug/mL
	ketamine-D4	100ug/mL
6	Norketamine	100ug/mL
	Norketamine-D4	100ug/mL
7	Cocaine	100ug/mL
	Cocaine-D3	100ug/mL
8	Benzoylecgonine	100ug/mL
	Benzoylecgonine-D3	100ug/mL
9	3,4-Methylenedioxyamphetamine	100ug/mL
	3,4-Methylenedioxyamphetamine-D4	100ug/mL
10	3,4-methylenedioxymethamphetamine	100ug/mL
	3,4-methylenedioxymethamphetamine -D4	100ug/mL
11	Cathinone	100ug/mL
	Cathinone-D5	100ug/mL
12	Methcathinone	100ug/mL
	Methcathinone-D5	100ug/mL
13	Fentanyl	100ug/mL
	Fentanyl-D5	100ug/mL
14	Diazepam	100ug/mL
	Diazepam-D5	100ug/mL
15	Estazolam	100ug/mL
	Estazolam-D5	100ug/mL
16	methadone	100ug/mL
	methadone-D10	100ug/mL
17	N-(1-methoxy-3,3-dimethyl-1-oxobutan-2-yl)-1-(5-fluoropentyl)- 1H-indole-3-carboxamide(5F-MDMB-PICA)	100ug/mL
	N-(1-methoxy-3,3-dimethyl-1-oxobutan-2-yl)-1-(5-fluoropentyl)-1H-indole- 3-carboxamide-D4	100ug/mL
